# Supplementary material for: Socioeconomic inequalities in sport participation: pattern per sport and time trends – a repeated cross-sectional study
Source: BMC Public Health. 2023 Apr 28;23:785. doi: 10.1186/s12889-023-15650-7 (PMC10141913; doi:10.1186/s12889-023-15650-7)
Supplement: Supplementary file 1 — Supplementary Material 1 [file 12889_2023_15650_MOESM1_ESM.pdf]

SUPPLEMENTARY INFORMATION

BMC Public Health

Socioeconomic inequalities in sport participation: pattern per sport and time trends – A repeated cross-sectional study

Viviane Richard MSc.[a], Giovanni Piumatti PhD.[b], Nick Pullen PhD.[a], Elsa Lorthe R.M., PhD.[a], Idris Guessous MD., PhD.[c,d], Nicola Cantoreggi PhD.[e], Silvia Stringhini PhD.[a,f]

a. Unit of Population Epidemiology, Division of Primary Care Medicine, Geneva University Hospitals, Geneva, Switzerland
b. Fondazione Agnelli, Turin, Italy
c. Department of Health and Community Medicine, Faculty of Medicine, University of Geneva, Geneva, Switzerland
d. Division and Department of Primary Care Medicine, Geneva University Hospitals, Geneva, Switzerland
e. Institute of Global Health, University of Geneva, Geneva, Switzerland.
f. University Center for General Medicine and Public Health, University of Lausanne, Switzerland

CORRESPONDING AUTHOR

Silvia Stringhini
silvia.stringhini@hcuge.ch
Unité d'épidémiologie populationnelle
Rue Jean-Violette 29
1205 Genève
Switzerland
+41 22 305 58 61

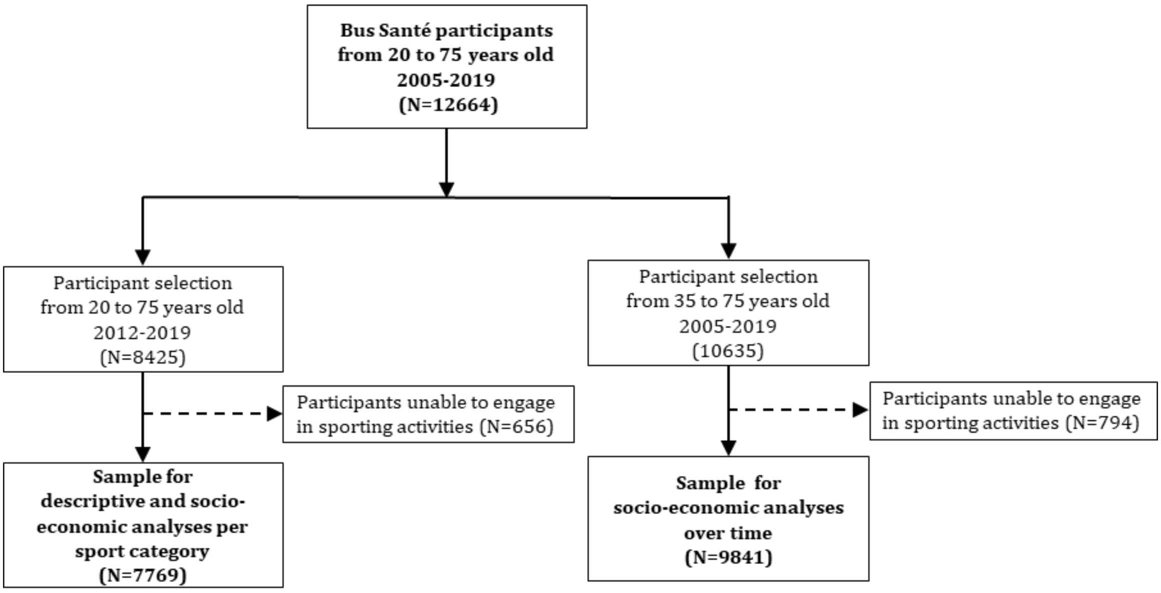

Supplementary 1: Participant's selection process

Supplementary 2: Relative index of inequality (RII) of the educational level, household income and occupational position among adults aged 20 to 75 years old, stratified by sport category and sex

	All				Men				Women			
	Minimally adj.[a]		Fully adj.[b]		Minimally adj.[a]		Fully adj.[b]		Minimally adj.[a]		Fully adj.[b]	
**Sport category[c]**	**RII**	**CI (95%)**	**RII**	**CI (95%)**	**RII**	**CI (95%)**	**RII**	**CI (95%)**	**RII**	**CI (95%)**	**RII**	**CI (95%)**
**All**												
Educational level	1.78	1.64-1.92	1.56	1.45-1.69	1.77	1.59-1.98	1.55	1.39-1.73	1.77	1.58-1.98	1.56	1.39-1.74
Household income	1.61	1.50-1.73	1.45	1.35-1.55	1.65	1.49-1.81	1.48	1.34-1.63	1.57	1.42-1.74	1.40	1.27-1.56
Occupational position	1.60	1.47-1.74	1.46	1.35-1.59	1.68	1.50-1.88	1.54	1.38-1.73	1.50	1.32-1.71	1.35	1.19-1.54
**Individual[c]**												
Educational level	1.83	1.64-2.03	1.56	1.40-1.74	1.83	1.59-2.12	1.54	1.33-1.77	1.81	1.55-2.12	1.58	1.34-1.85
Household income	1.70	1.55-1.88	1.51	1.37-1.66	1.66	1.46-1.89	1.47	1.29-1.67	1.76	1.52-2.04	1.55	1.33-1.79
Occupational position	1.77	1.58-1.98	1.58	1.41-1.77	1.89	1.63-2.19	1.69	1.46-1.96	1.60	1.33-1.93	1.42	1.18-1.71
**Racket[c]**												
Educational level	3.69	2.41-5.64	3.18	2.06-4.88	5.34	3.13-9.10	4.66	2.73-7.94	1.68	0.82-3.43	1.35	0.64-2.82
Household income	2.87	1.98-4.16	2.50	1.71-3.66	3.65	2.38-5.62	3.21	2.07-4.98	1.50	0.72-3.14	1.22	0.57-2.63
Occupational position	2.78	1.87-4.13	2.45	1.64-3.68	3.69	2.34-5.82	3.37	2.13-5.34	1.02	0.37-2.79	0.71	0.24-2.08
**Group[c]**												
Educational level	1.79	1.53-2.09	1.58	1.35-1.86	1.48	1.14-1.92	1.31	1.00-1.70	2.01	1.66-2.44	1.77	1.45-2.16
Household income	1.36	1.18-1.57	1.22	1.05-1.41	1.17	0.92-1.49	1.03	0.80-1.32	1.49	1.25-1.78	1.34	1.12-1.61
Occupational position	1.37	1.14-1.65	1.27	1.05-1.52	1.10	0.83-1.44	1.02	0.77-1.35	1.65	1.30-2.09	1.52	1.19-1.93
**Special[c]**												
Educational level	4.47	3.20-6.25	3.81	2.71-5.38	4.78	3.07-7.42	4.19	2.67-6.58	4.18	2.51-6.97	3.41	2.01-5.79
Household income	4.34	3.25-5.80	3.70	2.75-4.98	5.23	3.58-7.64	4.51	3.05-6.66	3.45	2.19-5.42	2.94	1.85-4.67
Occupational position	4.06	2.88-5.74	3.69	2.59-5.24	4.31	2.78-6.70	3.99	2.55-6.25	3.98	2.24-7.07	3.55	1.98-6.38

[a] Generalized linear model following a quasi-Poisson distribution, adjusted for age, sex, an interaction between age and sex, and country of birth. [b] Generalized linear model following a quasi-Poisson distribution, adjusted for age, sex, an interaction between age and sex, country of birth, smoking, BMI, self-perceived health and presence of chronic disease. [c] Sport categories: Individual (running, brisk walking, racing bicycle, strength training/weightlifting, swimming, ice-/roller-skating); Racket (tennis/badminton, squash); Group (dance, football, handball, gymnastics, judo/karate); Special (golf, downhill/water skiing, cross-country skiing, diving)

Supplementary 3: Slope index of inequality (SII) of the educational level, household income and occupational position among adults aged 20 to 75 years old, stratified by sport category and sex

	All				Men				Women			
	Minimally adj.[a]		Fully adj.[b]		Minimally adj.[a]		Fully adj.[b]		Minimally adj.[a]		Fully adj.[b]	
**Sport category[c]**	**SII**	**CI (95%)**	**SII**	**CI (95%)**	**SII**	**CI (95%)**	**SII**	**CI (95%)**	**SII**	**CI (95%)**	**SII**	**CI (95%)**
**All**												
Educational level	0.33	0.29-0.37	0.26	0.21-0.30	0.33	0.27-0.40	0.26	0.19-0.32	0.33	0.26-0.39	0.26	0.19-0.32
Household income	0.28	0.24-0.32	0.22	0.18-0.26	0.30	0.24-0.36	0.24	0.18-0.30	0.27	0.21-0.32	0.20	0.14-0.26
Occupational position	0.28	0.23-0.33	0.23	0.18-0.28	0.32	0.25-0.38	0.27	0.20-0.33	0.24	0.16-0.31	0.18	0.10-0.25
**Individual[c]**												
Educational level	0.25	0.21-0.30	0.19	0.14-0.23	0.27	0.21-0.33	0.19	0.13-0.25	0.24	0.17-0.30	0.18	0.12-0.24
Household income	0.23	0.19-0.27	0.18	0.14-0.22	0.23	0.17-0.29	0.18	0.12-0.24	0.23	0.17-0.29	0.18	0.12-0.24
Occupational position	0.26	0.21-0.31	0.21	0.15-0.26	0.30	0.23-0.37	0.25	0.18-0.32	0.19	0.12-0.27	0.14	0.07-0.22
**Racket[c]**												
Educational level	0.06	0.04-0.08	0.05	0.03-0.07	0.11	0.08-0.14	0.10	0.07-0.14	0.01	-0.01-0.03	0.01	-0.01-0.03
Household income	0.06	0.04-0.07	0.05	0.03-0.07	0.10	0.06-0.13	0.09	0.06-0.12	0.01	-0.01-0.03	0.01	-0.02-0.03
Occupational position	0.06	0.04-0.08	0.05	0.03-0.07	0.10	0.06-0.13	0.09	0.06-0.13	0.00	-0.03-0.03	-0.01	-0.04-0.02
**Group[c]**												
Educational level	0.15	0.11-0.19	0.12	0.08-0.16	0.08	0.03-0.13	0.05	0.00-0.11	0.21	0.15-0.27	0.17	0.12-0.23
Household income	0.08	0.04-0.12	0.05	0.01-0.09	0.03	-0.02-0.08	0.01	-0.04-0.06	0.13	0.07-0.18	0.09	0.04-0.15
Occupational position	0.07	0.03-0.12	0.06	0.01-0.10	0.02	-0.04-0.07	0.00	-0.05-0.06	0.14	0.08-0.21	0.12	0.05-0.19
**Special[c]**												
Educational level	0.11	0.09-0.14	0.10	0.08-0.12	0.14	0.10-0.18	0.13	0.09-0.17	0.09	0.06-0.11	0.07	0.04-0.10
Household income	0.12	0.10-0.15	0.11	0.09-0.13	0.16	0.13-0.20	0.15	0.11-0.19	0.08	0.05-0.11	0.07	0.04-0.10
Occupational position	0.12	0.09-0.15	0.11	0.08-0.14	0.15	0.11-0.20	0.14	0.10-0.19	0.08	0.05-0.12	0.08	0.04-0.12

[a] Linear model adjusted for age, sex, an interaction between age and sex, and country of birth. [b] Linear model adjusted for age, sex, an interaction between age and sex, country of birth, smoking, BMI, self-perceived health and presence of chronic disease. [c] Sport categories: Individual (running, brisk walking, racing bicycle, strength training/weightlifting, swimming, ice-/roller-skating); Racket (tennis/badminton, squash); Group (dance, football, handball, gymnastics, judo/karate); Special (golf, downhill/water skiing, cross-country skiing, diving).

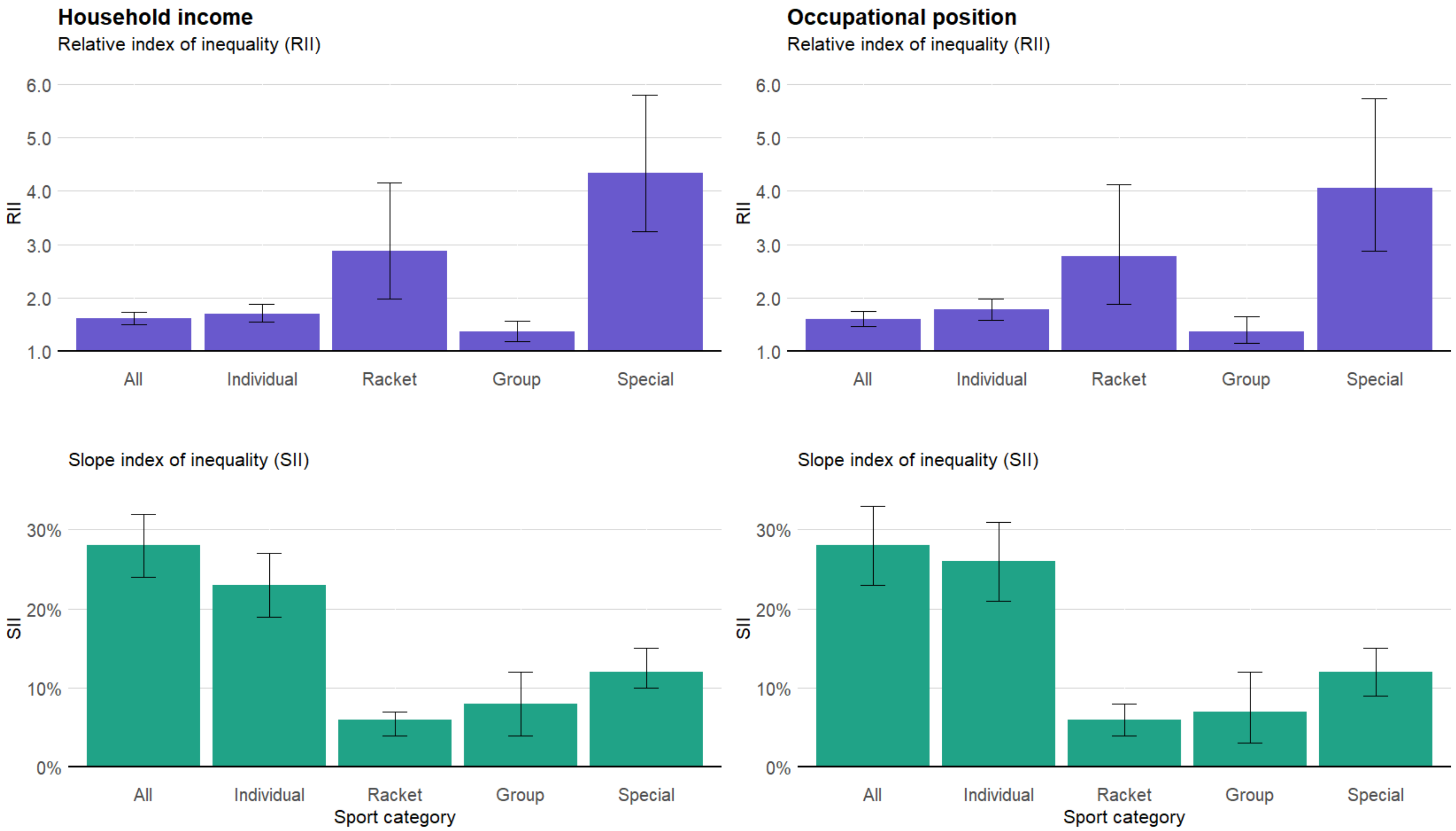

Supplementary 4: Relative and slope indexes of inequality (RII/SII) and 95% confidence intervals of sport participation among Geneva adults aged 20 to 75 years old stratified by sport category. Generalized linear model following a quasi-Poisson distribution for the RII and linear model for the SII. Minimal model, adjusted for age, sex, an interaction between age and sex, and country of birth. Sport categories: Individual (running, brisk walking, racing bicycle, strength training/weightlifting, swimming, ice-/roller-skating); Racket (tennis/badminton, squash); Group (dance, football, handball, gymnastics, judo/karate); Special (golf, downhill/water skiing, cross-country skiing, diving). N=6925 for household income and n=5428 for occupational position.

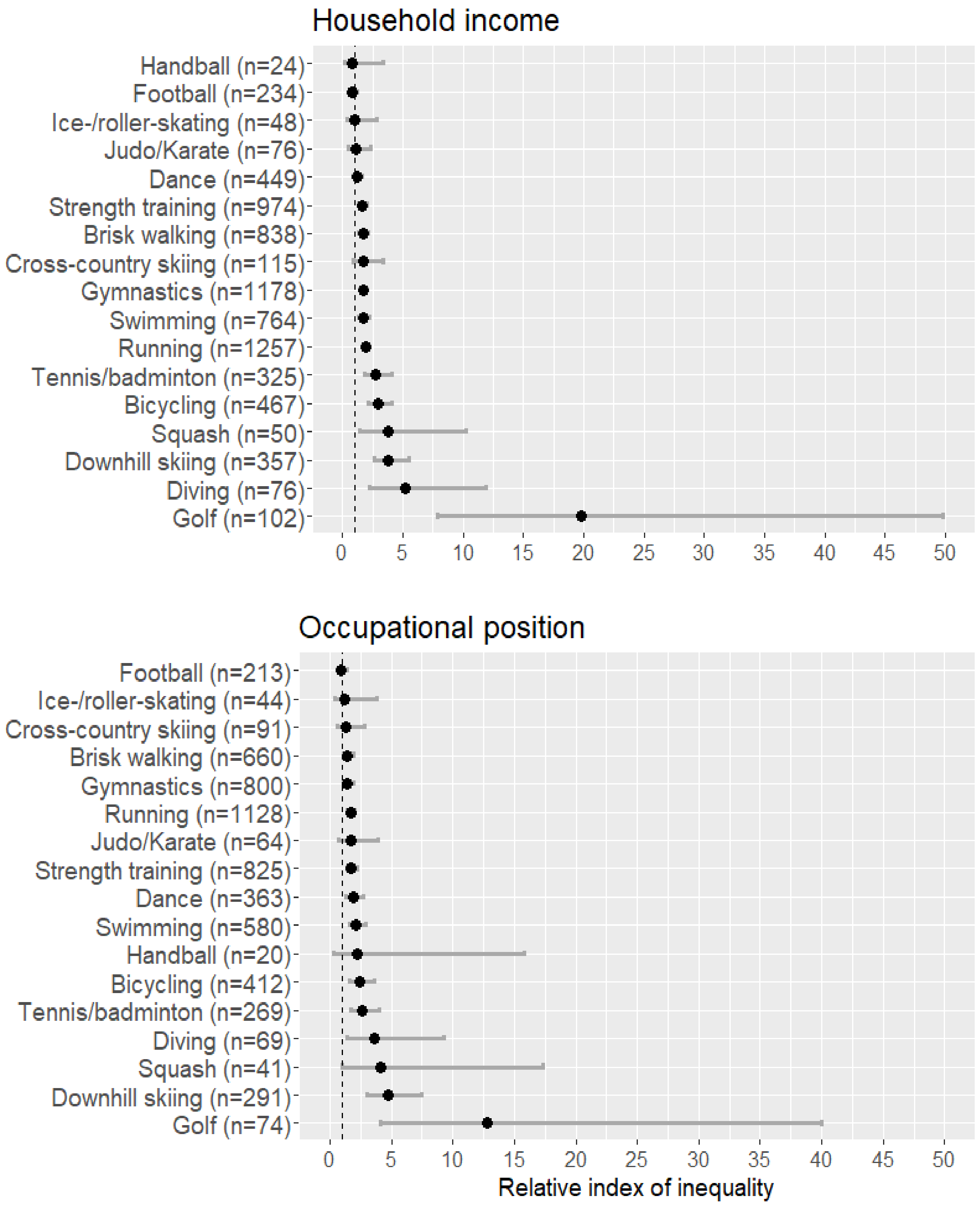

Supplementary 5: Relative index of inequality (RII), stratified by specific sport among Geneva adults aged 20 to 75 years old. Generalized linear model following a quasi-Poisson distribution adjusted for age, sex, an interaction between age and sex, and country of birth. N=6925 for household income and n=5428 for occupational position.

Supplementary 6: Yearly change of the relative and slope index of inequality (RII/SII) of sport participation from 2005 to 2019 among adults aged 35 to 75 years old, and sensitivity analysis from 2008 to 2019

	**All**				**Men**				**Women**				**35-54 years old**				**55-75 years old**			
	**Minimally adj.[c]**		**Fully adj.[d]**		**Minimally adj.[c]**		**Fully adj.[d]**		**Minimally adj.[c]**		**Fully adj.[d]**		**Minimally adj.[c]**		**Fully adj.[d]**		**Minimally adj.[c]**		**Fully adj.[d]**	
	**β**	**p-value**	**β**	**p-value**	**β**	**p-value**	**β**	**p-value**	**β**	**p-value**	**β**	**p-value**	**β**	**p-value**	**β**	**p-value**	**β**	**p-value**	**β**	**p-value**
**2005-2019**																				
**Relative index of inequality [a]**																				
Educational level	1.01	0.184	1.02	0.119	1.02	0.180	1.02	0.348	1.01	0.421	1.02	0.155	1.00	0.808	1.00	0.955	**1.04**	**0.024**	**1.04**	**0.013**
Household income	1.02	0.064	1.01	0.281	1.01	0.618	0.99	0.444	**1.03**	**0.028**	**1.03**	**0.024**	1.00	0.807	0.98	0.200	**1.04**	**0.006**	**1.05**	**0.003**
Occupational position	1.01	0.412	1.00	0.794	1.01	0.454	1.00	0.913	1.01	0.665	1.01	0.575	1.02	0.158	1.01	0.479	0.99	0.806	0.99	0.575
**Slope index of inequality [b]**																				
Educational level	0.01	0.099	0.01	0.067	0.01	0.169	0.01	0.242	0.01	0.317	0.01	0.150	0.00	0.545	0.00	0.813	**0.02**	**0.017**	**0.02**	**0.008**
Household income	**0.01**	**0.024**	0.01	0.148	0.01	0.405	0.00	0.684	**0.02**	**0.012**	**0.02**	**0.013**	0.00	0.535	-0.01	0.292	**0.02**	**0.003**	**0.02**	**0.002**
Occupational position	0.01	0.248	0.00	0.587	0.01	0.309	0.00	0.849	0.01	0.589	0.01	0.666	0.01	0.072	0.01	0.291	0.00	0.869	-0.01	0.598
**2008-2019**																				
**Relative index of inequality [a]**																				
Educational level	1.01	0.404	1.01	0.409	1.01	0.632	1.00	0.847	1.01	0.380	1.02	0.281	1.00	0.978	0.99	0.648	**1.03**	**0.082**	**1.04**	**0.037**
Household income	1.01	0.167	1.01	0.442	1.00	0.999	0.98	0.394	**1.03**	**0.033**	**1.03**	**0.045**	1.00	0.860	0.98	0.276	**1.04**	**0.029**	**1.05**	**0.008**
Occupational position	1.01	0.336	1.00	0.748	1.01	0.585	1.00	0.911	1.02	0.379	1.00	0.829	1.02	0.178	1.01	0.563	1.00	0.961	0.99	0.834
**Slope index of inequality [b]**																				
Educational level	0.01	0.330	0.01	0.317	0.00	0.681	0.00	0.777	0.01	0.308	0.01	0.254	0.00	0.781	0.00	0.724	**0.02**	**0.090**	**0.02**	**0.032**
Household income	0.01	0.097	0.01	0.283	0.00	0.802	-0.01	0.573	**0.02**	**0.021**	**0.02**	**0.032**	0.00	0.615	-0.01	0.357	**0.02**	**0.027**	**0.02**	**0.007**
Occupational position	0.01	0.244	0.00	0.587	0.01	0.460	0.00	0.732	0.01	0.332	0.00	0.801	0.01	0.097	0.01	0.384	0.00	0.980	0.00	0.841

β : yearly change of the RII/SII (exponential of the coefficient of the socioeconomic indicator x year term for the RII; coefficient of the socioeconomic indicator x year term for the SII). Results in bold indicate a p-value < 0.05.

[a] Generalized linear model following a quasi-Poisson distribution; [b] Linear model; [c] Adjusted for socioeconomic indicator, age, sex, country of birth, year, and interactions: age x sex, socioeconomic indicator x year, age x year, sex x year, country of birth x year; [d] Adjusted for socioeconomic indicator, age, sex, country of birth, smoking, BMI, self-perceived health, presence of chronic disease and interactions: year, age x sex, socioeconomic indicator x year, age x year, sex x year, country of birth x year, smoking x year, BMI x year, self-perceived health x year, presence of chronic disease x year.

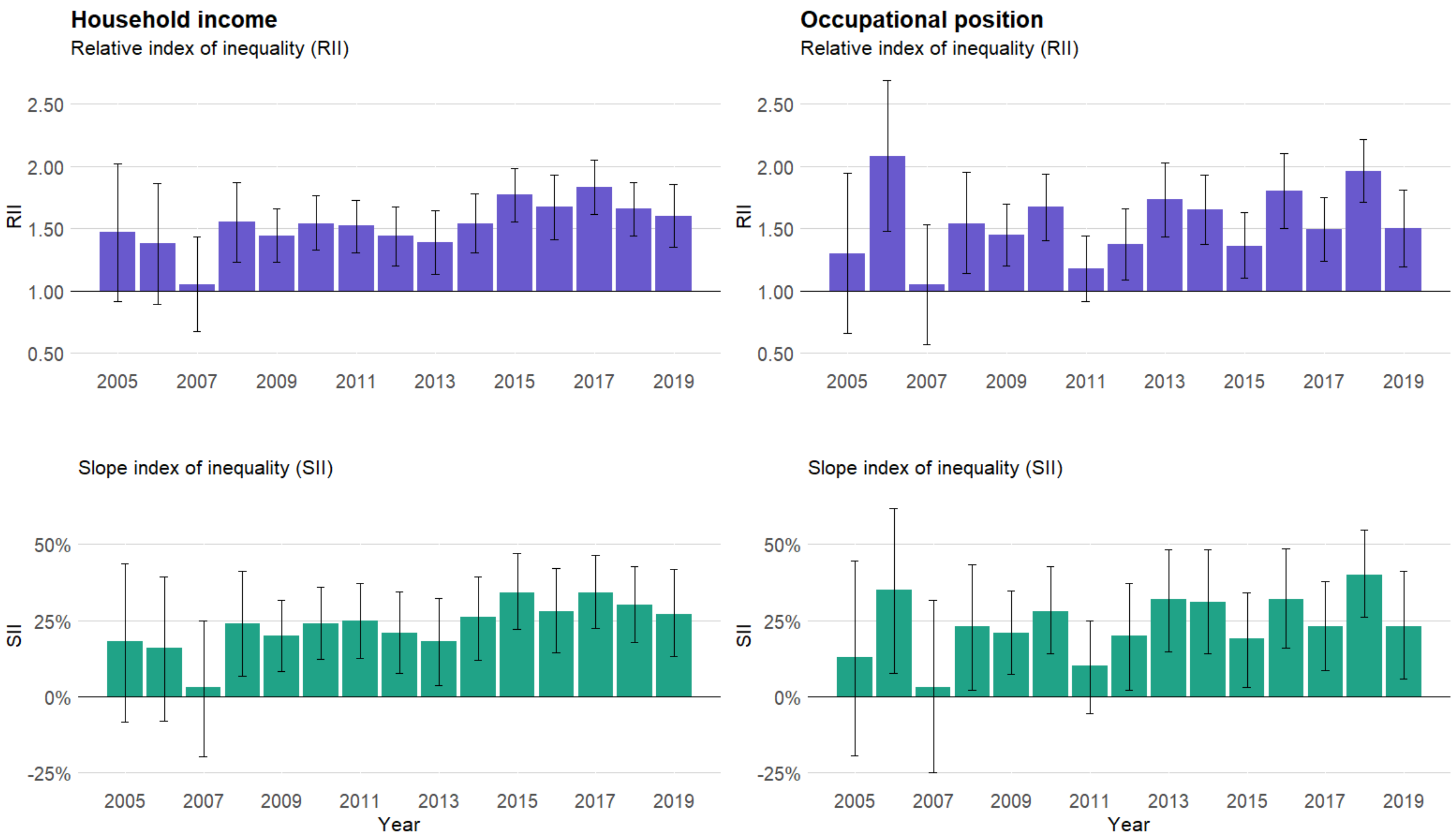

Supplementary 7: Relative and slope indexes of inequality (RII/SII) and 95% confidence intervals of sport participation, per year, among Geneva adults aged 35 to 75 years old. Generalized linear model following a quasi-Poisson distribution for the RII and linear model for the SII. Minimal model adjusted for sex, age, an interaction between age and sex, and country of birth. N=9251 for household income and n=6967 for occupational position.
